# Association of the *CTLA4* Gene with Graves' Disease in the Chinese Han Population

**DOI:** 10.1371/journal.pone.0009821

**Published:** 2010-03-23

**Authors:** Shuang-Xia Zhao, Chun-Ming Pan, Huang-Ming Cao, Bing Han, Jing-Yi Shi, Jun Liang, Guan-Qi Gao, Yong-De Peng, Qing Su, Jia-Lun Chen, Jia-Jun Zhao, Huai-Dong Song

**Affiliations:** 1 Ruijin Hospital, State Key Laboratory of Medical Genomics, Molecular Medicine Center, Shanghai Institute of Endocrinology, Shanghai Jiao Tong University (SJTU) School of Medicine, Shanghai, China; 2 Department of Endocrinology, Fourth Hospital of Xuzhou, Xuzhou, Jiangsu, China; 3 Department of Endocrinology, The People's Hospital of Linyi, Linyi, Shandong, China; 4 Department of Endocrinology, The First People's Hospital, Shanghai Jiao Tong University (SJTU) School of Medicine, Shanghai, China; 5 Department of Endocrinology, Xin Hua Hospital, Shanghai Jiao Tong University (SJTU) School of Medicine, Shanghai, China; 6 Department of Endocrinology, Shandong Province Hospital, Shandong University, Jinan, Shandong, China; Innsbruck Medical University, Austria

## Abstract

To determine whether genetic heterogeneity exists in patients with Graves' disease (GD), the *cytotoxic T-lymphocyte associated 4* (*CTLA-4*) gene, which is implicated a susceptibility gene for GD by considerable genetic and immunological evidence, was used for association analysis in a Chinese Han cohort recruited from various geographic regions. Our association study for the SNPs in the *CTLA4* gene in 2640 GD patients and 2204 control subjects confirmed that *CTLA4* is the susceptibility gene for GD in the Chinese Han population. Moreover, the logistic regression analysis in the combined Chinese Han cohort revealed that SNP rs231779 (allele frequencies *p* = 2.81×10^−9^, OR = 1.35, and genotype distributions *p* = 2.75×10^−9^, OR = 1.42) is likely the susceptibility variant for GD. Interestingly, the logistic regression analysis revealed that SNP rs35219727 may be the susceptibility variant to GD in the Shandong population; however, SNP, rs231779 in the *CTLA4* gene probably independently confers GD susceptibility in the Xuzhou and southern China populations. These data suggest that the susceptibility variants of the *CTLA4* gene varied between the different geographic populations with GD.

## Introduction

Graves' disease, which affects 1.2% of western populations (0.5% clinical and 0.7% subclinical) [1] and 0.25–1.09% of the Chinese population [2], is an autoimmune disorder in which the body produces auto-antibodies to the receptor for thyroid-stimulating hormone (TSH), leading to hyperthyroidism. Although environmental agents, such as infection [3] and stress, are undoubtedly important in the development of Graves' disease in susceptible individuals, it has been estimated in twin studies that around 80% of the predisposition to GD is due to genetic factors [4]. However, similar to other common complex diseases, the identification of the susceptibility gene for GD has been challenging. Recently, genome-wide association studies (GWAS) have uncovered the susceptibility genes of some common diseases [5]–[8]. However, variability between studies in the measured significance of the validated loci has appeared, and has suggested that genetic heterogeneity exists in type 2 diabetes [5]–[8]. In a recent whole genome linkage study by Tomer Y., distinct genes were suggested to predispose to autoimmune thyroid diseases (AITD) in different subsets of patients [9]. Most recently, our data have shown that, similar to most Mendelian monogenic disorders, the susceptibility variants of a gene that predisposes to GD varied among patients from different geographic populations [10]. The goal of the present study was to confirm that genetic heterogeneity exists in patients with GD.

Many genetic approaches, including candidate gene association studies [10]–[18] and GWAS [19] have been applied to identify genetic variants predisposing to GD, and several genes have been proposed as candidates, including *Fc receptor-like 3* (*FCRL3*) [12],*CD40* [14], *human leukocyte antigen* (*HLA*) [15], *cytotoxic T lymphocyte antigen 4* (*CTLA-4*) [16], [18], *protein tyrosine phosphatase, non-receptor type 22* (*PTPN22*) [17], *thyroid-stimulating hormone receptor* (*TSHR*) [11], [20], *the small antisense transcript of zinc-finger gene* (*SAS-ZFAT*) [13], and *Secretoglobin Family 3A Member 2* (*SCGB3A2*) [10]. However, most of these candidate susceptibility genes have been proven to be controversial. Only the *HLA* region on chromosome 6p21 [11], [19] and the *CTLA-4* gene on chromosome 2q33 [21]–[28] have been extensively studied, and considerable genetic and immunological evidence has suggested that they are important for GD. Furthermore, several SNPs, such as A49G, CT60, JO31 in the CTLA-4 gene, rather than in CD28 gene have been revealed association with Graves' disease [21]–[28].

In the present work, in order to confirm that *CTLA4* gene is associated with GD, and to ask whether the susceptibility variants in the gene differed among the different geographic populations with GD, SNPs in *CTLA4* gene were selected for genotyping in a Chinese Han cohort containing 2640 patients with GD and 2204 control subjects.

## Results

### Association analysis of the *CTLA4* gene in a combined Chinese Han population

**Figure 1:**
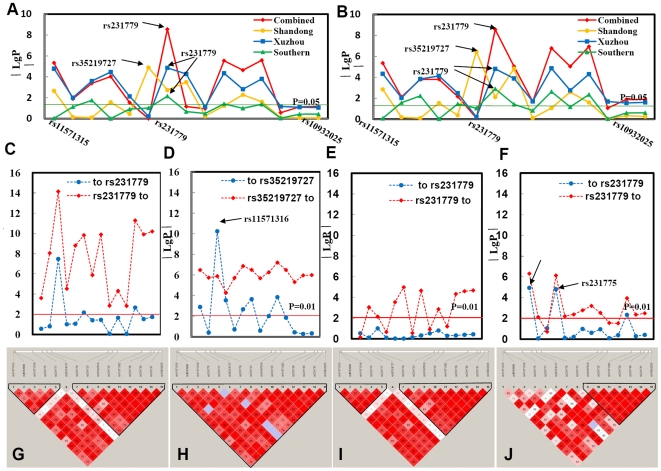
The results of association analysis of SNPs in the *CTLA4* gene region from different populations. A total of 47 SNPs located in the *CTLA4* gene region were genotyped in all subjects from different geographic regions of China. After removal of the SNPs with minor allele frequencies (MAF) <1%, missing data above 20% or HWE *p*≤1×10^−6^ in the controls, the allele frequencies (**A**) and genotype distributions (**B**) in GD and control subjects from different populations were plotted [−log10(*p* value) against location]. The most significantly associated SNPs in the combined Chinese Han, Xuzhou, and southern populations were all located at rs231779, with the smallest *p* values of 2.81×10^−9^, 1.37×10^−5^, 0.0071 in allele frequency difference (**A**) and 2.75×10^−9^, 1.55×10^−5^, 0.0013 in genotype distributions (**B**), respectively. In the Shandong population the most significantly locus was located at rs35219727, with the smallest *p* values of 1.30×10^−5^ in allele frequencies (**A**) and 3.57×10^−7^ in genotype distributions (**B**) (see Tables 1 and 2 for detailed information). **C**–**F**: Two-locus logistic regression analyses of rs231779 in the combined Chinese Han population (**C**), rs35219727 in the Shandong population (**D**), rs2321779 in the Xuzhou population (**E**) and rs231779 in the Southern China population (**F**). SNPs rs231779 and rs35219727were put individually into the regression models as the best makers, and all other markers were sequentially added to see if a second locus could improve the model. In the combined Chinese Han population, three of the 14 SNPs suitable for logistic regression analysis hurt the model with rs231779 (**C**), at the *p* value <0.01. In contrast, we tested a regression model by taking each of 14 loci in turn and adding the test locus to it. All the markers could be improved by adding rs231779 (**C**). At the same time, in the Shandong population, rs35219727 improved the model with each of the other 14 SNPs; however, seven SNPs hurt the model with rs35219727, except SNP rs11571316 (**D**) (see Table 3 for detailed information). In the Xuzhou and Southern China populations, most of the models with other SNPs could be improved by adding SNP rs231779, however, no or only two SNPs can improve the model with rs231779 (**E, F**). **G–J**: The SNPs linkage disequilibrium (LD) region for the Combined Chinese Han (**G**), Shandong (**H**), Xuzhou (**I**) and the southern China (**J**) populations in the *CTLA4* gene region were analyzed with haploview software.

Forty-seven SNPs in the *CTLA4* gene region were selected for genotyping in the Chinese Han cohort, containing 2640 patients with GD and 2204 control subjects, which were recruited from different geographic regions of China. Among the 47 SNPs, 44 SNPs with call rates of more than 80% were further analyzed in 2640 GD patients and 2204 control subjects. Of those, 17 SNPs with unique alleles and five SNPs with minor allele frequencies (MAF) of less than 1% were removed from the association analysis. In addition, seven SNPs with Hardy-Weinberg equilibrium (HME) of *p*≤1×10^−6^ in controls were also eliminated from the analysis [29]. Finally, 15 of the 47 SNPs in the *CTLA4* gene region were included in the association analysis (Table S1). The allele frequencies (Table 1) and the genotype distributions (Table 2) for these 15 SNPs were analyzed in 2640 GD patients and 2204 control subjects from different geographic regions of China. And all samples were analyzed in the same lab and under the same conditions. Out of the 15 SNPs, eight SNPs have significantly different allele frequencies and genotype distributions (at *p*-value <0.001 level) between the GD and normal subjects and the strongest association was measured for one SNP in the first intron, rs231779 (allele frequencies *p* = 2.81×10^−9^, OR = 1.35, 95%CI = 1.23–1.48 and genotype distributions *p* = 2.75×10^−9^, OR = 1.42, 95%CI = 1.27–1.60) (Tables 1 and 2, Fig. 1A and 1B). It was interesting that rs231775 (i.e., A49G polymorphism in exon 1 of the *CTLA4* gene) and rs11571302 (i.e., JO31 polymorphism in the 3′ untranslated region (UTR) of the *CTLA4* gene), which have been reported to be susceptibility loci of GD, also showed significant differences between GD patients and controls in the combined Chinese Han population (rs231775: allele frequencies *p* = 9.39×10^−5^, OR = 1.24, 95%CI = 1.12–1.38 and genotype distributions *p* = 0.0002, OR = 1.28, 95%CI = 1.13–1.45, rs11571302: allele frequencies *p* = 2.29×10^−5^, OR = 1.26, 95%CI = 1.14–1.40 and genotype distributions *p* = 9.26×10^−6^, OR = 1.31, 95%CI = 1.16–1.47, respectively) (Tables 1 and 2, Fig. 1A and 1B).

**Table 1:** Allele frequencies in GD and control subjects for SNPs in the CTLA4 gene region in different populations.

Populations		Combined					Shandong				
SNP	Allele	control (%)	case (%)	p Value	OR	OR (95%CI)	control (%)	case (%)	p Value	OR	OR (95%CI)
**rs11571315**	**A**	3063 (71.8)	3890 (76.3)	**4.65×10^−6^**	1.26	1.15–1.39	948 (71.2)	1407 (76.4)	**0.0022**	1.31	1.12–1.54
	**G**	1201 (28.2)	1206 (23.7)				384 (28.8)	435 (23.6)			
**rs4553808**	**A**	3304 (86.7)	4274 (88.6)	**0.0127**	1.19	1.05–1.36	1109 (88.9)	1575 (88.4)	0.7068	0.95	0.76–1.20
	**G**	506 (13.3)	548 (11.4)				139 (11.1)	207 (11.6)			
**rs11571316**	**C**	3048 (80.3)	3714 (83.5)	**0.0004**	1.25	1.11–1.39	984 (79.1)	1106 (79.6)	0.7842	1.03	0.85–1.24
	**T**	748 (19.7)	732 (16.5)				260 (20.9)	284 (20.4)			
**rs231775**	**G**	2713 (70.5)	2790 (74.8)	**9.39×10^−5^**	1.24	1.12–1.38	849 (71.2)	783 (75.7)	**0.0271**	1.26	1.04–1.52
	**A**	1135 (29.5)	938 (25.2)				343 (28.8)	251 (24.3)			
**rs231777**	**C**	3320 (86.1)	4294 (87.8)	**0.0288**	1.16	1.03–1.32	1109 (88.7)	1603 (87.5)	0.3449	0.89	0.71–1.11
	**T**	536 (13.9)	596 (12.2)				141 (11.3)	229 (12.5)			
**rs35219727**	**G**	3500 (92.0)	4485 (91.9)	0.9792	1.00	0.85–1.17	1207 (96.9)	1693 (92.9)	**1.30×10^−5^**	0.42	0.29–0.61
	**A**	306 (8.0)	393 (8.1)				39 (3.1)	129 (7.1)			
**rs231779**	**T**	2903 (69.2)	3837 (75.2)	**2.81×10^−9^**	1.35	1.23–1.48	875 (70.2)	1409 (75.7)	**0.0019**	1.32	1.12–1.55
	**C**	1293 (30.8)	1267 (24.8)				371 (29.8)	453 (24.3)			
**rs231723**	**G**	3457 (72.4)	3674 (76.5)	**2.55×10^−5^**	1.24	1.13–1.36	945 (70.9)	1455 (75.9)	**0.0038**	1.29	1.10–1.51
	**A**	1317 (27.6)	1130 (23.5)				387 (29.1)	463 (24.1)			
**rs10197010**	**A**	3259 (84.6)	4193 (86.0)	0.1129	1.11	0.99–1.25	1087 (87.1)	1562 (86.3)	0.5549	0.93	0.75–1.15
	**C**	591 (15.4)	685 (14.0)				161 (12.9)	248 (13.7)			
**rs231725**	**A**	2431 (63.0)	3329 (68.2)	**2.86×10^−6^**	1.26	1.15–1.38	799 (64.0)	1231 (67.6)	0.0602	1.17	1.01–1.36
	**G**	1427 (37.0)	1553 (31.8)				449 (36.0)	591 (32.4)			
**rs11571302**	**C**	3336 (77.8)	4246 (81.6)	**2.29×10^−5^**	1.26	1.14–1.40	992 (77.0)	1563 (81.4)	**0.0052**	1.31	1.10–1.55
	**A**	952 (22.2)	958 (18.4)				296 (23.0)	357 (18.6)			
**rs231729**	**A**	2718 (63.2)	3547 (68.2)	**2.61×10^−6^**	1.25	1.15–1.36	824 (63.9)	1298 (68.0)	**0.0244**	1.20	1.04–1.40
	**T**	1580 (36.8)	1653 (31.8)				466 (36.1)	610 (32.0)			
**rs231730**	**T**	3745 (86.5)	4494 (87.4)	0.2671	1.08	0.95–1.21	1166 (87.4)	1638 (87.0)	0.7745	0.97	0.78–1.19
	**A**	583 (13.5)	650 (12.6)				168 (12.6)	244 (13.0)			
**rs231731**	**T**	3662 (85.0)	4496 (86.4)	0.0735	1.12	1.00–1.26	1111 (85.6)	1644 (85.9)	0.8254	1.02	0.84–1.25
	**C**	648 (15.0)	710 (13.6)				187 (14.4)	270 (14.1)			
**rs10932025**	**G**	3663 (85.1)	4510 (86.6)	0.0677	1.12	1.00–1.26	1119 (86.2)	1657 (86.5)	0.8383	1.02	0.83–1.26
	**C**	639 (14.9)	700 (13.4)				179 (13.8)	259 (13.5)			

Xuzhou					Southern				
control (%)	case (%)	p Value	OR	OR (95%CI)	control (%)	case (%)	p Value	OR	OR (95%CI)
1098 (67.3)	1251 (74.6)	**1.65×10^−5^**	1.43	1.23–1.66	1017 (78.2)	1232 (78.1)	0.9253	0.99	0.83–1.18
534 (32.7)	425 (25.4)				283 (21.8)	346 (21.9)			
1345 (85.1)	1463 (88.5)	**0.0099**	1.34	1.09–1.64	850 (86.6)	1236 (89.2)	0.0735	1.28	1.00–1.64
235 (14.9)	191 (11.5)				132 (13.4)	150 (10.8)			
1334 (81.6)	1456 (86.7)	**0.0003**	1.46	1.21–1.76	730 (79.5)	1152 (83.7)	**0.0178**	1.32	1.07–1.64
300 (18.4)	224 (13.3)				188 (20.5)	224 (16.3)			
1082 (67.6)	1245 (74.7)	**3.47×10^−5^**	1.42	1.22–1.65	782 (74.1)	762 (74.1)	0.9726	1.00	0.83–1.11
518 (32.4)	421 (25.3)				274 (25.9)	266 (25.9)			
1356 (83.9)	1457 (87.5)	**0.0074**	1.34	1.10–1.63	855 (86.4)	1234 (88.6)	0.1223	1.23	0.96–1.58
260 (16.1)	209 (12.5)				135 (13.6)	158 (11.4)			
1339 (85.4)	1412 (86.1)	0.5995	1.06	0.87–1.29	954 (96.2)	1380 (97.5)	0.0961	1.53	0.96–2.43
229 (14.6)	228 (13.9)				38 (3.8)	36 (2.5)			
1100 (68.7)	1237 (76.1)	**1.37×10^−5^**	1.45	1.24–1.70	928 (68.8)	1191 (73.7)	**0.0071**	1.27	1.08–1.49
502 (30.3)	389 (23.9)				420 (31.2)	425 (26.3)			
1098 (67.5)	1249 (74.4)	**5.37×10^−5^**	1.40	1.20–1.63	1414 (77.9)	970 (80.3)	0.1384	1.16	0.97–1.39
528 (32.5)	429 (25.6)				402 (22.1)	238 (19.7)			
1330 (82.4)	1411 (84.9)	0.0747	1.20	1.00–1.45	842 (85.2)	1220 (86.8)	0.3192	1.14	0.90–1.44
284 (17.6)	251 (15.1)				146 (14.8)	186 (13.2)			
1010 (62.3)	1159 (69.7)	**4.50×10^−5^**	1.39	1.20–1.60	622 (62.8)	939 (67.3)	**0.0383**	1.22	1.02–1.44
610 (37.7)	505 (30.3)				368 (37.2)	457 (32.7)			
1293 (79.3)	1392 (84.0)	**0.0015**	1.36	1.14–1.63	1051 (76.7)	1291 (79.4)	0.1022	1.17	0.98–1.39
337 (20.7)	266 (16.0)				319 (23.3)	335 (20.6)			
1017 (62.5)	1148 (69.2)	**0.0002**	1.35	1.17–1.56	877 (63.6)	1101 (67.4)	**0.0417**	1.18	1.02–1.38
611 (37.5)	510 (30.8)				503 (36.4)	533 (32.6)			
1356 (83.1)	1436 (85.6)	0.0688	1.21	1.00–1.46	1223 (89.8)	1420 (89.6)	0.9032	0.98	0.78–1.25
276 (16.9)	242 (14.4)				139 (10.2)	164 (10.4)			
1358 (83.1)	1411 (85.5)	0.0801	1.20	0.99–1.45	1193 (86.6)	1441 (87.8)	0.3702	1.11	0.90–1.38
276 (16.9)	239 (14.5)				185 (13.4)	201 (12.2)			
1353 (83.0)	1417 (85.4)	0.0874	1.19	0.99–1.44	1191 (86.7)	1436 (87.9)	0.3623	1.11	0.90–1.38
277 (17.0)	243 (14.6)				183 (13.3)	198 (12.1)			

The *p* value with bold letters indicate those allele frequencies with significant differences between GD and normal subjects.

**Table 2:** Genotype distributions of the SNPs in the CTLA4 gene region in GD patients and controls in different populations.

Populations		Combined					Shandong				
SNP	Genotype	control (%)	case (%)	p Value	OR	OR (95%CI)	control (%)	case (%)	p Value	OR	OR (95%CI)
**rs11571315**	**AA**	1109 (52.0)	1496 (58.7)	**4.39×10^−6^**	1.31	1.17–1.47	336 (50.5)	539 (58.5)	**0.0014**	1.39	1.13–1.69
	**AG+GG**	1023 (48.0)	1052 (41.3)				330 (49.5)	382 (41.5)			
**rs4553808**	**AA**	1430 (75.1)	1893 (78.5)	**0.0075**	1.21	1.05–1.40	492 (78.8)	694 (77.9)	0.6569	0.95	0.74–1.21
	**AG+GG**	475 (24.9)	518 (21.5)				132 (21.2)	197 (22.1)			
**rs11571316**	**CC**	1221 (64.3)	1553 (69.9)	**0.0002**	1.29	1.13–1.46	383 (61.6)	433 (62.3)	0.7863	1.03	0.83–1.29
	**CT+TT**	677 (35.7)	670 (30.1)				239 (38.4)	262 (37.7)			
**rs231775**	**GG**	945 (49.1)	1030 (55.3)	**0.0002**	1.28	1.13–1.45	301 (50.5)	295 (57.1)	**0.0287**	1.30	1.03–1.65
	**GA+AA**	979 (50.9)	834 (44.7)				295 (49.5)	222 (42.9)			
**rs231777**	**CC**	1423 (73.8)	1891 (77.3)	**0.0068**	1.21	1.05–1.39	490 (78.4)	702 (76.6)	0.4170	0.90	0.71–1.15
	**CT+TT**	505 (26.2)	554 (22.7)				135 (21.6)	214 (23.4)			
**rs35219727**	**GG**	1602 (84.2)	2046 (83.9)	0.7917	0.98	0.83–1.15	586 (94.1)	782 (85.8)	**3.57×10^–7^**	0.38	0.26–0.56
	**GA+AA**	301 (15.8)	393 (16.1)				37 (5.9)	129 (14.2)			
**rs231779**	**TT**	1026 (48.9)	1471 (57.6)	**2.75×10^−9^**	1.42	1.27–1.60	318 (51.0)	539 (57.9)	**0.0078**	1.32	1.08–1.62
	**TC+CC**	1072 (51.1)	1081 (42.4)				305 (49.0)	392 (42.1)			
**rs231723**	**GG**	1252 (52.5)	1413 (58.8)	**8.99×10^−6^**	1.30	1.16–1.45	227 (33.7)	413 (44.5)	**1.51×10^−5^**	1.57	1.28–1.93
	**GA+AA**	1135 (47.5)	989 (41.2)				446 (66.3)	516 (55.5)			
**rs10197010**	**AA**	1368 (71.1)	1810 (74.2)	**0.0204**	1.17	1.02–1.34	470 (75.3)	676 (74.7)	0.7818	0.97	0.76–1.22
	**AC+CC**	557 (28.9)	629 (25.8)				154(24.7)	229 (25.3)			
**rs231725**	**AA**	751 (38.9)	1143 (46.8)	**1.71×10^−7^**	1.38	1.22–1.56	254 (40.7)	411 (45.1)	0.0868	1.20	0.97–1.47
	**AG+GG**	1178 (61.1)	1298 (53.2)				370 (59.3)	500 (54.9)			
**rs11571302**	**CC**	1293 (60.3)	1731 (66.5)	**9.26×10^−6^**	1.31	1.16–1.47	379 (58.9)	636 (66.3)	**0.0026**	1.37	1.12–1.69
	**CA+AA**	851 (39.7)	871 (33.5)				265 (41.1)	324 (33.8)			
**rs231729**	**AA**	843 (39.2)	1219 (46.9)	**1.16×10^−7^**	1.37	1.22–1.54	261 (40.5)	440 (46.1)	**0.0253**	1.26	1.03–1.54
	**AT+TT**	1306 (60.8)	1381 (53.1)				384 (59.5)	514 (53.9)			
**rs231730**	**TT**	1617 (74.7)	1977 (76.9)	0.0858	1.12	0.98–1.28	507 (76.0)	717 (76.2)	0.9322	1.01	0.80–1.27
	**TA+AA**	547 (25.3)	595 (23.1)				160 (24.0)	224 (23.8)			
**rs231731**	**TT**	1544 (71.6)	1948 (74.8)	**0.0132**	1.18	1.03–1.34	470 (72.4)	709 (74.1)	0.4582	1.09	0.87–1.36
	**TC+CC**	611 (28.4)	655 (25.2)				179 (27.6)	248 (25.9)			
**rs10932025**	**GG**	1549 (72.0)	1959 (75.2)	**0.0129**	1.18	1.04–1.34	478 (73.7)	718 (74.9)	0.5590	1.07	0.85–1.34
	**GC+CC**	602 (28.0)	646 (24.8)				171 (26.3)	240 (25.1)			

Xuzhou					Southern				
control (%)	case (%)	p Value	OR	OR (95%CI)	control (%)	case (%)	p Value	OR	OR (95%CI)
373 (45.7)	467 (55.7)	**4.62×10^−5^**	1.49	1.23–1.81	400 (61.5)	490 (62.1)	0.8261	1.02	0.83–1.27
443 (54.3)	371 (44.3)				250 (38.5)	299 (37.9)			
573 (72.5)	646 (78.1)	**0.0092**	1.35	1.08–1.70	365 (74.3)	553 (79.8)	**0.0266**	1.36	1.04–1.79
217 (27.5)	181 (21.9)				126 (25.7)	140 (20.2)			
553 (67.7)	639 (76.1)	**0.0001**	1.52	1.22–1.88	285 (62.1)	481 (69.9)	**0.0059**	1.42	1.11–1.82
264 (32.3)	201 (23.9)				174 (37.9)	207 (30.1)			
370 (46.3)	467 (56.1)	**7.32×10^−5^**	1.48	1.22–1.80	274 (51.9)	268 (52.1)	0.9366	1.01	0.79–1.29
430 (53.8)	366 (43.9)				254 (48.1)	246 (47.9)			
567 (70.2)	638 (76.6)	**0.0033**	1.39	1.12–1.73	366 (73.9)	551 (79.2)	**0.0347**	1.34	1.02–1.76
241 (29.8)	195 (23.4)				129 (26.1)	145 (20.8)			
557 (71.0)	592 (72.2)	0.6098	1.06	0.85–1.31	459 (92.5)	672 (94.9)	0.0892	1.50	0.94–2.42
227 (29.0)	228 (27.8)				37 (7.5)	36 (5.1)			
391 (48.8)	484 (59.5)	**1.55×10^−5^**	1.54	1.27–1.88	317 (47.0)	448 (55.4)	**0.0013**	1.40	1.14–1.72
410 (51.2)	329 (40.5)				357 (53.0)	360 (44.6)			
374 (46.0)	465 (55.4)	**0.0001**	1.46	1.20–1.77	412 (63.7)	534 (68.9)	**0.0376**	1.26	1.01–1.58
439 (54.0)	374 (44.6)				235 (36.3)	241 (31.1)			
544 (67.4)	604 (72.7)	**0.0198**	1.29	1.04–1.59	354 (71.7)	530 (75.4)	0.1481	1.21	0.93–1.57
263 (32.6)	227 (27.3)				140 (28.3)	173 (24.6)			
311 (38.4)	408 (49.0)	**1.39×10^−5^**	1.54	1.27–1.88	186 (37.6)	324 (46.4)	**0.0024**	1.44	1.14–1.82
499 (61.6)	424 (51.0)				309 (62.4)	374 (53.6)			
517 (63.4)	586 (70.7)	**0.0018**	1.39	1.13–1.71	397 (58.0)	509 (62.6)	0.0666	1.21	0.99–1.50
298 (36.6)	243 (29.3)				288 (42.0)	304 (37.4)			
313 (38.5)	401 (48.4)	**5.00×10^−5^**	1.50	1.23–1.82	269 (39.0)	378 (46.3)	**0.0044**	1.35	1.10–1.66
501 (61.5)	428 (51.6)				421 (61.0)	439 (53.7)			
562 (68.9)	621 (74.0)	**0.0205**	1.29	1.04–1.59	548 (80.5)	639 (80.7)	0.9183	1.01	0.78–1.31
254 (31.1)	218 (26.0)				133 (19.5)	153 (19.3)			
562 (68.8)	608 (73.7)	**0.0280**	1.27	1.03–1.58	512 (74.3)	631 (76.9)	0.2504	1.15	0.91–1.45
255 (31.2)	217 (26.3)				177 (25.7)	190 (23.1)			
559 (68.6)	611 (73.6)	**0.0245**	1.28	1.03–1.58	512 (74.5)	630 (77.1)	0.2429	1.15	0.91–1.46
256 (31.4)	219 (26.4)				175 (25.5)	187 (22.9)			

The *p* value with bold letters indicate those genotype frequencies with significant differences between GD and normal subjects.

Meanwhile, the linkage disequilibrium (LD) regions of 15 SNPs within the *CTLA4* gene were evaluated using the Haploview program [30]. Two LD region composed of these SNPs were observed in the combined Chinese Han population and were located between SNPs rs11571315 and rs231777, and SNPs rs231779 and rs10932025, respectively (Fig. 1G).

Next, to identify the susceptibility variants of GD in the *CTLA4* gene region, the genotype data of 15 SNPs suitable for logistic regression analysis in the combined Chinese Han population were further mined by forward and two-locus logistic regression analysis [16], [31] (Table 3 and Fig. 1C). Forward logistic regression result suggested that rs11571316, rs231779, rs231725 and rs231730 were the independent susceptibility variants in the combined Chinese Han cohorts. Because no statistical difference in allele frequencies and genotype distributions between GD patients and healthy control was detected at SNP rs231730, the remained three SNPs rs11571316, rs231779 and rs231725 were further analyzed by two-locus regression analysis. Among them, rs231779 can improve all of the models with one of other 14 SNPs, with a cut-off p-value <0.01. Nevertheless, only three SNPs (rs11571316, rs35219727, and rs231730) could improve this model with SNP rs231779 (p = 3.36×10^−8^, 0.0069 and 0.0022, respectively). Among the three SNPs that could improve the model with rs231779, two SNPs (rs35219727 and rs231730) did not show a significant difference between the patients with GD and control subjects. Meanwhile, SNP rs11571316 in the promoter of the CTLA4 gene was located on one of the two LD blocks that did not include SNP rs231779 (Fig. 1G). Thus, in the combined Chinese Han population, with regard to the SNPs in the CTLA4 gene region, SNP rs231779 with the lowest p-value among 15 SNPs of CTLA4 was likely the most important SNP for the susceptibility to GD because it could improve the model with each one of other 14 SNPs. However, these results do not exclude the possibility that SNP rs231779 and rs11571316 act in combination to increase susceptibility to GD.

**Table 3:** The results of two-locus logistic regression analysis in different populations.

Populations	Combined						Shandong						Xuzhou		Southern	
SNP	to rs11571316	rs11571316 to	to rs231779	rs231779 to	to rs231725	rs231725 to	to rs35219727	rs35219727 to	to rs231779	rs231779 to	to rs231723	rs231723 to	to rs231779	rs231779 to	to rs231779	rs231779 to
rs11571315	**1.20E-11**	**7.27E-07**	0.2757	**0.0003**	0.1266	0.0645	**0.0013**	**3.32E-07**	0.6840	0.5462	0.9445	0.4596	0.2916	0.7156	**1.15E-05**	**4.88E-07**
rs4553808	0.0230	0.4255	0.1564	**8.99E-09**	0.5489	**7.77E-07**	0.3934	**1.81E-06**	0.0294	**0.0008**	0.0108	**4.30E-05**	0.7681	**0.0009**	0.8580	**0.0074**
rs11571316			**3.36E-08**	**7.19E-15**	**3.06E-06**	**9.26E-12**	**5.77E-11**	**1.30E-06**	**8.99E-19**	**3.13E-08**	**5.01E-19**	**4.43E-10**	0.0987	**0.0071**	0.0895	0.1937
rs231775	**2.08E-09**	**0.0002**	0.0980	**2.98E-05**	0.5171	**0.0084**	**0.0003**	**5.39E-05**	0.4237	0.6329	0.4993	0.7148	0.7851	0.2208	**1.53E-05**	**7.38E-07**
rs231777	0.0367	0.2481	0.0881	**1.60E-09**	0.4482	**4.03E-07**	0.1937	**1.94E-06**	**0.0068**	**0.0004**	**0.0026**	**2.31E-05**	0.9412	**0.0003**	0.7633	**0.0062**
rs35219727	0.0692	0.1378	**0.0069**	**1.51E-10**	**0.0044**	**8.63E-09**			**1.37E-07**	**0.0021**	**3.18E-07**	**0.0002**	0.9412	**1.04E-05**	0.6071	**0.0042**
rs231779	**7.19E-15**	**3.36E-08**			**0.0014**	0.8675	**0.0021**	**1.37E-07**			0.6216	0.3715				
rs231723	**1.32E-09**	**1.91E-05**	0.0395	**1.36E-06**	0.5802	**0.0041**	**0.0002**	**3.18E-07**	0.3715	0.6216			0.7135	0.2810	0.1042	**0.0017**
rs10197010	0.1880	0.1916	0.0359	**1.31E-10**	0.0793	**1.68E-08**	0.2539	**2.07E-06**	0.0178	**0.0008**	**0.0066**	**0.0001**	0.4741	**2.20E-05**	0.2465	**0.0006**
rs231725	**9.26E-12**	**3.06E-06**	0.8675	**0.0014**			**0.0097**	**5.53E-07**	0.6616	0.0733	0.4683	0.0194	0.2988	0.1223	0.1119	**0.0029**
rs11571302	**1.11E-16**	**9.28E-13**	0.0225	**4.94E-05**	0.0527	**0.0096**	**0.0001**	**6.26E-08**	0.1672	0.0743	0.1693	0.0468	0.1582	**0.0014**	0.8147	0.0276
rs231729	**6.27E-11**	**5.23E-06**	0.8802	**0.0015**	0.9110	0.4190	0.0139	**3.27E-07**	0.7089	0.0489	0.7507	0.0378	0.5083	0.0638	0.4148	0.0304
rs231730	0.5933	0.1864	**0.0022**	**5.27E-12**	0.0092	**1.65E-09**	0.3657	**4.88E-06**	0.0389	**0.0004**	0.0216	**0.0001**	0.4736	**4.65E-05**	**0.0046**	**0.0001**
rs231731	0.1579	0.2423	0.0300	**1.27E-10**	0.1148	**1.20E-07**	0.5227	**1.10E-06**	0.1293	**0.0010**	0.1247	**0.0004**	0.4100	**2.52E-05**	0.5233	**0.0044**
rs10932025	0.1794	0.2043	0.0187	**6.43E-11**	0.1108	**1.19E-07**	0.4629	**1.03E-06**	0.0700	**0.0005**	0.1093	**0.0003**	0.3674	**2.09E-05**	0.3895	**0.0033**

Bold *p* Values indicates *p*<0.01.

The false positive report probability (FPRP) of the SNPs with significant association to GD in the combined Chinese Han cohort was also analyzed. In the present study, the FPRP value was calculated for each genetic variant using the assigned prior probability range, the statistical power to detect an odds ratio of 1.5, and detected odds ratios and *p* values. As shown in Table 4, among the 10 genetic variants with a significant difference between the patients with GD and healthy individuals, the FPRP values of five SNPs were below 0.2 for the prior probability from 0.25 to 0.0001, which was a relatively high prior probability range. However, the FPRP values for rs231779 were very low even for low prior probabilities, since the FPRP value remained below 0.2 even for a prior probability of 0.00001 (Table 4). Interestingly, the case control study for these 10 SNPs with significant differences in allele frequencies between the 2640 patients with GD and 2204 control individuals has more than 99.5% statistical power to detect a SNP with an α level equal to their reported *p* value, corresponding to relative risks of 1.5 for GD (Table 4). Notably, the FPRP values of SNP rs11571316 (*p* = 0.0004), a possible susceptibility variant for GD in the Chinese Han population by logistic regression analysis, were below 0.2 just for the prior probability from 0.25 to 0.01; whereas, the values were more than 0.2 if the prior probability was less than 0.01, suggesting that the SNP rs11571316 may be a false positive report SNP.

**Table 4:** False positive report probability (FPRP) values for ten SNPs with significant difference between 2640 patients with GD and 2204 health individuals.

SNP	Odds ratio (95% CI)	Reported *p*-Value	Statistical power under recessive model^a^	Prior probability					
				0.25	0.1	0.01	0.001	0.0001	0.00001
**rs11571315**	1.26 (1.15–1.39)	4.65×10^−6^	0.9999	**1.40×10^−5^**	**4.19×10^−5^**	**0.0005**	**0.0046**	**0.0444**	0.3174
**rs4553808**	1.19 (1.05–1.36)	0.0127	0.9999	**0.0366**	**0.1024**	**0.5566**	**0.9268**	0.9922	0.9992
**rs11571316**	1.25 (1.11–1.39)	0.0004	0.9999	**0.0013**	**0.0038**	**0.0399**	**0.2957**	0.8078	0.9768
**rs231775**	1.24 (1.12–1.38)	9.39×10^−5^	0.9999	**0.0003**	**0.0008**	**0.0092**	**0.0857**	0.4841	0.9037
**rs231777**	1.16 (1.03–1.32)	0.0288	1.0000	**0.0795**	0.2057	0.7402	0.9664	0.9965	0.9997
**rs231779**	1.35 (1.23–1.48)	2.81×10^−9^	0.9956	**8.46×10^−9^**	**2.54×10^−8^**	**2.79×10^−7^**	**2.82×10^−6^**	**2.82×10^−5^**	**0.0003**
**rs231723**	1.24 (1.13–1.36)	2.55×10^−5^	1.0000	**7.65×10^−5^**	**0.0002**	**0.0025**	**0.0248**	0.2032	0.7183
**rs231725**	1.26 (1.15–1.38)	2.86×10^−6^	1.0000	**8.58×10^−6^**	**2.58×10^−5^**	**0.0003**	**0.0029**	**0.0278**	0.2225
**rs11571302**	1.26 (1.14–1.40)	2.29×10^−5^	0.9998	**6.88×10^−5^**	**0.0002**	**0.0023**	**0.0224**	**0.1866**	0.6964
**rs231729**	1.25 (1.15–1.36)	2.61×10^−6^	1.0000	**7.82×10^−6^**	**2.35×10^−5^**	**0.0003**	**0.0026**	**0.0254**	0.2068

a Statistical power is the power to detect an odds ratio of 1.5 for the homozygotes with the rare genetic variant, with an α level equal to the reported p-Value. FPRP values below 0.2 are in bold face.

### Association analysis in different geographic regions of the Chinese Han population

It was interesting that there was variability in the significance of 15 SNPs in the *CTLA4* gene region across different geographic regions of the Chinese Han population. In the Shandong population, there were seven SNPs exhibiting significantly different allele frequencies and genotype distributions between 970 GD patients and 682 control subjects, with *p*-values <0.05 (Tables 1 and 2, and Fig. 1A and 1B). Further analysis of these seven SNPs revealed that the most significant difference in allele frequencies and genotype distributions was detected at SNP rs35219727 (*p* = 1.30×10^−5^, OR 0.42, 95%CI 0.29–0.61 and *p* = 3.57×10^−7^, OR 0.38, 95%CI 0.26–0.56, respectively) (Tables 1 and 2, and Fig. 1A, 1B), which was also in intron 1 of the *CTLA4* gene. Next, these 15 SNPs in the Shandong population were further analyzed using forward and two-locus logistic regression analysis. Forward logistic regression result revealed that SNPs rs11571316, rs231777, rs35219727, rs231779 and rs231723 were the independent susceptibility variants. However, no statistical differences were detected at SNPs rs11571316 and rs231777 (Table 1, 2 and Fig. 1A, 1B). At the same time, the two-locus logistic regression result showed that rs35219727 could improve the model with each one of the other 14 SNPs; however, only seven SNPs could weakly improve the model with rs35219727, except SNP rs11571316 (Table 3 and Fig. 1D), with a cut-off *p*-value <0.01. Although SNP rs11571316 could significantly improve the model with rs35219727 in the Shandong population by two-locus logistic regression analysis, it showed no significant difference between patients with GD and control subjects (Tables 1, 2 and 3, and Fig. 1D). Interestingly, unlike Xuzhou and the combined Chinese Han population, only one LD region was discovered in the Shandong population by haploview analysis. This region was located between SNPs rs11571315 and rs10932025 (Fig. 1H). The results suggested that SNP rs35219727 was the susceptibility variant to GD in the Shandong population.

Meanwhile, in the Xuzhou population of 841 GD patients and 818 control subjects, out of 15 SNPs, 10 SNPs demonstrated different distribution patterns (at *p*-value <0.01 level) (Tables 1 and 2, and Fig. 1A and 1B). Among those 10 SNPs, five SNPs (rs11571315, rs231775, rs231779, rs231723, and rs231725) exhibited statistically significant differences in allele frequencies and genotype distributions, with the *p*-values <0.0001, and the most significant difference in the allele and genotype frequencies located at SNP rs231779 (*p* = 1.37×10^−5^, OR 1.45, 95%CI 1.24–1.70 and *p* = 1.55×10^−5^, OR 1.54, 95%CI 1.27–1.88) (Tables 1 and 2, and Fig. 1A and 1B). Notably, any two loci between these five SNPs (rs11571315, rs231775, rs231779, rs231723, and rs231725) were tightly linked in the Xuzhou population, and all D' values were greater than 97% (Fig. 1I). Forward logistic regression revealed that only SNP rs231779 was the independent susceptibility variant. At the same time, two-locus logistic regression analysis results showed that no SNP can improve the model with rs231779, however, rs231779 can improve the model with one of other nine out of 14 SNPs, with a cut-off *p* value <0.01 (Table 3). Interestingly, four out of five SNPs (rs11571315, rs231775, rs231723, and rs231725), which could not improve the model with rs231779, were strongly linked in the Xuzhou population and not the independent susceptibility variants in the forward logistic analysis. It was possible that SNP rs231779 conferred susceptibility to GD in the Xuzhou population. Haploview analysis results for the Xuzhou population revealed that there were two LD blocks located between SNPs rs11571315 and rs231777, and SNPs rs231779 and rs10932025, which were the same as the results from the combined Chinese Han population (Fig. 1I).

At the same time, association analysis was performed in 829 GD patients and 704 normal subjects from southern China, including Shanghai City and Fujian Province. We found that four out of 15 SNPs had allele frequencies of significant difference (Table 1) and seven SNPs had different genotype distributions (Table 2) between patients with GD and control subjects,with *p*-values <0.05. Among them, the locus with the most significant difference was located at SNP rs231779, and the *p*-values for allele frequencies and genotype distributions were 0.0071 and 0.0013, respectively (Tables 1 and 2). Forward logistic regression result suggested that SNPs rs11571315, rs231775 and rs231779 were the independent susceptibility variants. However, there were no statistical differences between GD patients and control subjects at SNPs rs11571315 and rs231775. Meanwhile, the two-locus logistic regression analysis in the southern China population revealed that rs231779 can improve the model with each of SNPs except three SNPs (rs11571316, rs11571302 and rs231729), however, only three SNPs (rs11571315, rs231775 and rs231730) can improve the model with SNP rs231779. Interestingly, the three SNPs improving the model with rs231779 did not show a significant difference between the patients with GD and control subjects recruited from southern China. Of note, SNPs rs11571316 and rs231779 were linked to each other (D' = 82%) in the southern China population by haploview analysis (Fig. 1J), but they did not influenced each other in the results of the two-locus logistic regression analysis (Table 3). Taken together these results suggested that SNP rs231779, in the *CTLA4* gene independently conferred susceptibility to GD in the southern China population.

## Discussion

Our case-control study of the SNPs in the *CTLA4* gene region from 2640 GD patients and 2204 control subjects verified that *CTLA4* is the susceptibility gene for GD in the Chinese Han population. Moreover, the logistic regression analysis in the combined Chinese Han cohorts revealed that SNP rs231779 is likely the susceptibility variant because it improved the model with any one of the other 14 SNPs in the *CTLA4* gene region. Interestingly, the FPRP value for SNP rs231779 was very low for the prior probability range and was quite robust even for low prior probabilities. These results suggest that rs231779 in intron 1 of *CTLA4* is associated with GD etiology in the combined Chinese Han population; although, it still remains possible that there are other susceptibility SNPs or gene(s) that cause the onset of GD. Similarly, SNP rs231779 was associated with susceptibility to GD in the Shanghai Han population of China with 436 patients with GD and 316 control subjects (allele frequency *p* = 0.013, OR 1.34, 95%CI 1.06–1.68 and genotype distribution *p* = 0.017, OR 1.44, 95%CI 1.07–1.93) [28] and in the Taiwan population with 208 Chinese GD patients and 171 healthy controls (genotype distribution *p* = 0.0008) [26].

The SNPs, rs231775 (Exon 1 +49G>A) [21]–[23], [26], [32] and rs3087243 (CT60) [16], [25], [28], have been previously reported to be the major susceptibility variant of GD in Europe Caucasian populations. In our present study, SNP rs231775, which was almost perfectly linked with rs231779 (D' value, 0.88∼0.97 in the combined Chinese Han and three different populations), showed a significant difference between GD patients and controls in the combined Chinese Han population, Shandong population and Xuzhou population (allele frequency *p* = 9.39×10^−5^, 0.0271 and 3.47×10^−5^, respectively). However, the logistic regression analysis suggested that the association signal of SNP rs231775 was accounted for the susceptibility variant SNP rs231779 in our combined Chinese Han population. Unfortunately, another SNP rs3087243 (CT60) was not in Hardy-Weinberg equilibrium (Table S1) and was removed from the final analysis, which was similar to the reported results from the Shanghai Chinese Han population [28]. Notably, SNP rs3087243 was strongly linked with SNPs rs231775 and rs231779 and the D' values were more than 0.95 in the Taiwan Chinese population [26]. Therefore, in our present study, SNP rs3087243 may be tagged by SNPs rs231775 and rs231779.

Although this study provides solid evidence for the association of the *CTLA4* gene with GD in our combined Chinese Han population, it was notable that the susceptibility variants of the *CTLA4* gene might vary in the patients with GD recruited from different geographic regions of China. In our case control analysis, the loci with the most significant associations to GD were located at SNP rs35219727 in the Shandong populations and rs231779 in the Xuzhou and southern China populations, respectively. Furthermore, the logistic regression analysis revealed that SNP rs35219727 might be the susceptibility variant of GD in the Shandong population. However, SNP rs231779 in the *CTLA4* gene probably independently conferred GD susceptibility in the combined Chinese Han population, Shandong and southern China populations. A recent and detailed study of a Japanese GD cohort investigated the SNPs across the TSHR region and identified single SNP associations with GD primarily located in intron 7 of TSHR [11]. However, in the more recent study by Brand, no evidence of an association of the TSHR intron 7 SNPs with GD in the UK European ancestry cohort was found [20]. Interestingly, their data demonstrate that the strongest signals of association with GD were within TSHR intron 1 in the UK European ancestry cohort [20]. Recently, two independently reports have shown that some intricately substructured is exists northern Han, central Han, and southern Han in the Chinese population. However, the genetic differentiation among these clusters are very small (FST _ 0.0002∼0.0009) [33], [34]. In the present study, the Xuzhou city is closed to Shandong province (subjects was mainly rectuited from Jinan and Linyi cities) and both of them belongs to the central Han population. In fact, the genetic differentiation is the most small in the central Han population in China [33], [34]. Thus, it is not obvious that the results were influenced by population substructure in the present study. These data, combined with our findings, suggest that the susceptibility variants of GD vary in populations from different geographic regions. More detailed analysis of different populations is needed to confirm this hypothesis.

## Materials and Methods

### Sample recruitment

A total of 2640 unrelated individuals with Graves' disease (GD) were recruited from different geographic regions of China. Among them, the numbers of GD individuals in Shandong Province, Xuzhou City, and southern China regions, including Shanghai and Fujian Province, were 970, 841, and 829, respectively. The control group was made up of 2204 unrelated healthy subjects from the same geographic region screened for the absence of thyroid disease. Within these subjects, 682, 818, and 704 control subjects were collected from Shandong, Xuzhou, and southern China. The diagnosis of GD was based on documented clinical and biochemical evidence of hyperthyroidism, diffused goiter, and the presence of at least one of the following items: positive TSH receptor antibody tests, diffusely increased ^131^I (iodine-131) uptake in the thyroid gland, or the presence of exophthalmos. All individuals classified as affected were interviewed and examined by experienced clinicians. All subjects were Chinese Han in origin. After receiving informed consent, 5-ml blood samples were collected from all participants for DNA preparations, as well as for biochemical measurements.

### Genotyping methodology, SNP selection, and quality control (QC) filters

All genotyping was performed using the Mass-Array™ Technology Platform of Sequenom, Inc (San Diego, California, USA). A total of 63 SNPs were identified in the *CTLA4* gene region from NCBI dbSNP (NCBI Human Genome Build 36.3). Subsequently, several procedures were taken for selecting these SNPs. Firstly, SNPs associated with GD in the previous reports and tag SNPs were selected. Secondly, SNPs were chosen with an average space of 50 bp. Finally, several SNPs were removed through the assembly of multiple-PCR primers. Accordingly, 47 SNPs in the *CTLA4* gene region in Supplementary Table S1 were genotyped for association analysis in 2640 GD individuals and 2204 normal subjects, collected from different geographic regions of China. Then several steps were taken for the SNPs quality control (QC) filters. Firstly, 22 of 47 SNPs with a unique allele or minor allele frequency (MAF) of less than 1% were removed from the association analysis. Secondly, rs41265961,rs3087245, and rs7565213 were removed from the analysis because of missing data above 20%. Finally, seven SNPs with Hardy-Weinberg equilibria (HME) *p*≤1×10^−6^ in controls were eliminated from the analysis [29].

### Statistical analysis of association

In the case-control design, allele/genotype frequencies, odds ratios (ORs), and significance values were analyzed by Chi-square analysis using SPSS (version 13.0; SPSS Inc). In order to exclude false positives, 20 neutral SNPs on different chromosomes were analyzed as genomic controls (GCs) and the GC inflation factor (λ_GC_) was 1.1734 [10]. All statistical results were normalized to the GC. A *p*-value <0.05 was considered significant. The genotype data were further mined by logistic regression analysis, as previously described [16], [31]. Linkage disequilibrium (LD) regions were analyzed by Haploview [30]. FPRP was analyzed using the FPRP calculation spreadsheet provided by Wacholder, et al [24]. The statistical power to detect an odds ratio of 1.5, with an α level equal to the reported *p*-value was also provided and the FPRP value for noteworthiness was preset to 0.5.

## Supporting Information

Table S1 (0.04 MB PDF)
