# Supplementary material for: Association of the CTLA4 Gene with Graves' Disease in the Chinese Han Population
Source: PLoS One. 2010 Mar 23;5(3):e9821. doi: 10.1371/journal.pone.0009821 (PMC2843719; doi:10.1371/journal.pone.0009821)
Supplement: Table S1 — (0.04 MB PDF) [file pone.0009821.s001.pdf]

Table S1 47 SNPs selected in CTLA4 gene region from the NCBI dbSNP and quality control filters

SNP	Description	Allele
rs35832561[1]	5' near gene	G
**rs11571315**	5' near gene	**A/G**
**rs4553808**	5' near gene	**A/G, -1661**
**rs11571316**	5' near gene	**C/T**
rs231772[1]	5' near gene	G
rs231773[1]	5' near gene	A
rs11571317[1]	5' near gene	C
rs2317741[1]	5' near gene	A
rs5742909[3]	5' near gene	C/T, -318
**rs231775**	exon_1	**A/G, +49G>A**
rs231776[1]	intron_1	G
**rs231777**	intron_1	**C/T**
rs231778[1]	intron_1	A
**rs35219727**	intron_1	**A/G**
**rs231779**	intron_1	**C/T**
rs35703452[1]	intron_1	DEL
rs1065442[1]	exon_2	T
rs41265959[1]	intron_2	T
rs41265961[2]	intron_2	G/A
rs231780[1]	intron_2	A
rs11571324[1]	intron_2	G
rs34162447[3]	exon_3	A/G
rs13384548[1]	exon_3	G
rs35411154[1]	exon_3	G
rs231721[1]	3' near gene	T
rs3087243[3]	3' near gene	A/G, CT60
rs231722[1]	3' near gene	T
**rs231723**	3' near gene	**A/G**
rs35902238[1]	3' near gene	DEL
**rs10197010**	3' near gene	**A/C**
rs3087245[2]	3' near gene	C/T
**rs231725**	3' near gene	**A/G**
rs34227960[1]	3' near gene	DEL
rs231726[3]	3' near gene	**C/T**
rs10932024[1]	3' near gene	T
rs3087246[3]	3' near gene	C/T
rs231727[3]	3' near gene	A/G
**rs11571302**	3' near gene	**C/A, JO31**
rs35541017[1]	3' near gene	DEL
rs35720565[1]	3' near gene	C
rs7565213[2]	3' near gene	A/G, JO30
**rs231729**	3' near gene	**A/T**
**rs231730**	3' near gene	**T/A**
rs5837885[1]	3' near gene	DEL
**rs231731**	3' near gene	**C/T**
**rs10932025**	3' near gene	**G/C**
rs11571297[3]	3' near gene	A/G, JO27

Note: [1, 2,3] showed the SNPs loci were removed from the analysis respectively because of MAF <1%, missing data above 20% and Hardy-Weinberg Equilibrium (HWE) $p \leqslant 1\times10^{-6}$ in controls.
